# A Multicenter Investigation of Minimally Invasive Sample Processing and the Pre-Analytic Assessment of NSCLC Small Biopsy Specimens for Molecular Testing

**DOI:** 10.3390/diagnostics16070962

**Published:** 2026-03-24

**Authors:** Anzi Tan, Lixi Huang, Liwen Jiang, Yingying Gu, Ping He, Zeyun Lin, Shiqi Tang, Chunping Liu, Mengshi Li, Zhucheng Chen, Yuying Chen, Jiangyu Zhang, Juhong Jiang

**Affiliations:** 1 State Key Laboratory of Respiratory Disease, National Clinical Research Center for Respiratory Disease, Guangzhou Institute of Respiratory Health, The First Affiliated Hospital of Guangzhou Medical University, Guangzhou 510120, China; tananzi99@163.com (A.T.); gyblgyy@126.com (Y.G.); linzeyun2021@126.com (Z.L.); pinyin77@163.com (S.T.); 13610123890@163.com (C.L.); limengshi93@163.com (M.L.); czp1249193405@163.com (Z.C.); 15119209474@163.com (Y.C.); 2 Department of Pathology, Sun Yat-Sen University Cancer Center, Guangzhou 510060, China; huanglx@sysucc.org.cn; 3 State Key Laboratory of Oncology in South China, Collaborative Innovation Center for Cancer Medicine, Guangzhou 510060, China; 4 Department of Pathology, Affiliated Cancer Hospital, Guangzhou Medical University, Guangzhou 510095, China; 15951157199@163.com; 5 Department of Pathology, The First Affiliated Hospital of Guangzhou Medical University, Guangzhou 510163, China; hp5567@163.com; 6 Department of Clinical Laboratory, Guangzhou Development District Hospital, Guangzhou 510730, China

**Keywords:** non-small cell lung carcinoma, ancillary testing, mutation detection, small biopsy, fixative medium, cell pellet

## Abstract

**Objectives**: In the era of precision oncology, the management of lung cancer depends fundamentally on the acquisition of sufficient neoplastic material for both definitive histological subtyping and comprehensive molecular profiling. This study aimed to investigate molecular testing adequacy rates for small lung biopsy specimens obtained via minimally invasive procedures at three high-volume oncology centers. Recognizing that a significant subset of specimens remains insufficient for analysis, we evaluated the utility of cell pellets derived from residual fixative media as a supplemental resource for ancillary molecular testing. **Methods**: Over a six-month period, specimen handling workflows for small biopsies were assessed across three high-volume oncology centers. The pre-analytic molecular adequacy of formalin-fixed paraffin-embedded (FFPE) tissue sections from patients diagnosed with non-small cell lung cancer (NSCLC) was evaluated. During the final two months of the study, in cases where the primary FFPE tissue was deemed inadequate for molecular profiling, the residual fixative solution was recovered and processed to generate supplemental cell pellets. **Results**: Using adequacy thresholds of >200 tumor cells per section and a tumor cell fraction (TCF) of ≥10% or ≥5% (depending on specific assay requirements), the overall adequacy rates for FFPE samples were 80.6% (2986/3705) and 88.9% (3293/3705), respectively. During the final two months, 18.9% (154/816) of cases exhibited inadequate FFPE sections. However, of these cases, 56% (86/154) yielded adequate cell pellets based on cellularity evaluation and DNA quantification. These results indicate that cell pellets collected from the fixative medium of thoracic small biopsies are a valuable supplemental material for ancillary testing. **Conclusions**: This multi-center investigation demonstrates that a notable subset of NSCLC specimens obtained via minimally invasive biopsy remains insufficient for molecular analysis. Cell pellet samples obtained from residual fixative media serve as a critical supplemental resource, effectively increasing the success rate of molecular adequacy in clinical practice.

## 1. Introduction

Molecular testing-guided targeted therapies have dramatically transformed the prognostic landscape for patients with non-small cell lung carcinoma (NSCLC). With the rapid discovery of novel oncogenic drivers, clinical guidelines for newly diagnosed advanced NSCLC continue to expand the requisite repertoire of therapeutic biomarkers [1,2,3]. Although advancements in minimally invasive techniques have facilitated specimen collection with reduced morbidity, the resulting samples are frequently limited in volume. These scant specimens must support a multifaceted diagnostic workflow, including definitive histological diagnosis, ancillary immunohistochemical (IHC) staining, and comprehensive molecular profiling. This competing demand for limited material presents a formidable challenge in clinical practice. Indeed, studies indicate that 10% to 30% of small biopsies are inadequate for downstream molecular analysis due to low tumor cellularity or insufficient DNA yield [4,5,6,7,8,9,10]. Consequently, a significant subset of patients either loses the opportunity for targeted intervention or must undergo the inherent risks of repeat biopsy. While liquid biopsies utilizing circulating tumor DNA (ctDNA) provide an alternative for tissue-limited cases, their clinical utility is often hindered by suboptimal sensitivity compared to tissue-based assays [11,12,13].

The most prevalent minimally invasive techniques in lung cancer diagnostics include Transbronchial forceps lung biopsy (TBLB) with or without concurrent endobronchial ultrasound-guided transbronchial needle aspiration (EBUS-TBNA), and CT- or ultrasound-guided transthoracic core needle biopsy (CNB) with or without concurrent fine-needle aspiration (FNA). Pathologically, TBLB and CNB yield histological small specimens, whereas EBUS-TBNA and FNA provide cytological aspirates.

Data from a 2021 Canadian study indicated that 10% of NSCLC samples were insufficient for molecular testing, and even among tissue-adequate samples, 16% yielded insufficient DNA [4]. Similarly, a retrospective analysis at MD Anderson Cancer Center found aggregate molecular adequacy rates of 84%, with FNA and EBUS-TBNA performing lower (72% and 77%, respectively) than CNB (90%) [5]. Conversely, Hendry et al. reported an 89.5% adequacy rate when integrating rapid on-site evaluation (ROSE), though success rates still varied by modality: 96.8% for CNB, 85.8% for FNA, and 88.8% for EBUS-TBNA [6].

Preliminary histological evaluation of hematoxylin and eosin (H&E) stained formalin-fixed paraffin-embedded (FFPE) tissue sections indicates that lung cancer specimens obtained via small biopsy are inherently fragile, frequently shedding microscopic tissue fragments or cell clusters into the fixative medium [14,15]. This phenomenon is particularly pronounced in poorly differentiated or extensively necrotic tumors. Unlike resilient normal stroma, neoplastic tissue is highly susceptible to fragmentation under the mechanical compression of biopsy forceps and the needle aspiration process. Furthermore, mechanical agitation during transport facilitates the shedding of poorly adherent neoplastic cells into the fixative solution, while stromal components remain structurally intact. At present, pathological workflows frequently neglect the diagnostic potential of tumor cells suspended in specimen fixatives. Routine processing focuses exclusively on the retrieval of macroscopically visible tissue for paraffin embedding, whereas microscopic clusters or fragments—too small for manual manipulation—remain unrecovered. As a result, this residual fixative medium, despite its potential cellular richness, is typically disposed of as medical waste.

Our previous research established a robust methodology for salvaging cell pellets from this residual fixative for molecular profiling [14,15]. A critical finding of our study is that the majority of lung biopsies yield high-quality tumor cell pellets within the residual medium. By integrating these pellets with traditional formalin-fixed paraffin-embedded (FFPE) tissue, we significantly improved the diagnostic adequacy rate. Our preliminary single-center study demonstrated that integrating a specialized collection workflow to salvage shed tumor cells from the fixative significantly improves the molecular success rate for histological specimens. The present project aims to validate the efficacy of these refined methods in improving molecular adequacy for lung cancer small biopsies across multiple centers.

## 2. Materials and Methods

### 2.1. Study Design and Patient Selection

This multicenter study enrolled patients diagnosed with non-small cell lung cancer (NSCLC) via conventional histopathological examination across three institutions: the First Affiliated Hospital of Guangzhou Medical University (Center A), Affiliated Cancer Hospital & Institute of Guangzhou Medical University (Center B), and Sun Yat-sen University Cancer Center (Center C). The study protocol was approved by the Institutional Review Board of the First Affiliated Hospital of Guangzhou Medical University (Ref: 2021-70; 16 August 2021).

Eligible patients underwent minimally invasive diagnostic procedures—including TBLB, CNB, and EBUS-TBNA—between January and June 2024. We prospectively and retrospectively monitored the handling workflows for small biopsy and cytological specimens at each center. The evaluation focused on the macroscopic state of specimens upon arrival at the pathology department, technician grossing methodologies, the presence of residual tissue in the fixative post-processing, and the overall pre-analytic adequacy of samples for downstream molecular analysis. Comprehensive clinicopathological data, including age, sex, specimen source, sampling technique, and IHC profiles, were retrieved from each institution’s clinical and pathological information systems.

### 2.2. TBLB Specimen Processing Methodologies

At each participating center, bronchoscopy unit support staff prepared sterilized 1 cm × 1 cm filter paper segments for specimen collection. During the TBLB procedure, two to five tissue fragments were typically collected and adhered to these segments before immersion in specimen bottles containing 10% neutral-buffered formalin.

**Figure 1:**
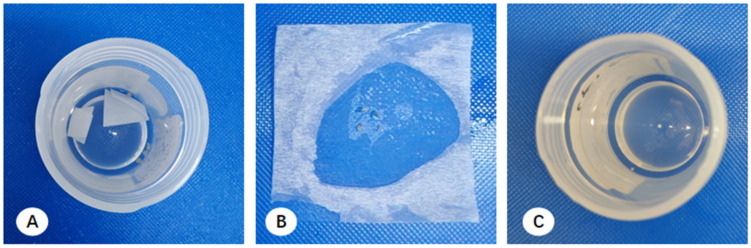
Presentation and processing of transbronchial lung biopsy (TBLB) specimens. (**A**) Initial state of TBLB specimens upon arrival at the pathology department; tissue fragments are partially adhered to filter paper segments or dispersed within the fixative. (**B**) Macroscopic processing: a pathology technician utilizes tweezers to retrieve all visible tissue fragments from both the filter paper and the fixative, transferring them onto embedding paper. (**C**) Post-processing status of the fixative; residual micro-fragments, which are too small for manual retrieval, remain visible in the solution.

Upon arrival at the pathology department, macroscopic assessment revealed multiple filter paper segments within the fixative; while some tissue remained adhered, other fragments had detached and were suspended freely. Pathologists used tweezers to scrape tissue from the paper and retrieve visible free-floating fragments, which were then wrapped in embedding paper for paraffin embedding. However, this manual recovery was limited to macroscopically visible fragments. Consequently, minute, ungraspable tissue residues consistently remained in the residual formalin across all centers, representing a systematic loss of diagnostic material (Figure 1).

### 2.3. CNB Specimen Processing Methodologies

**Figure 2:**
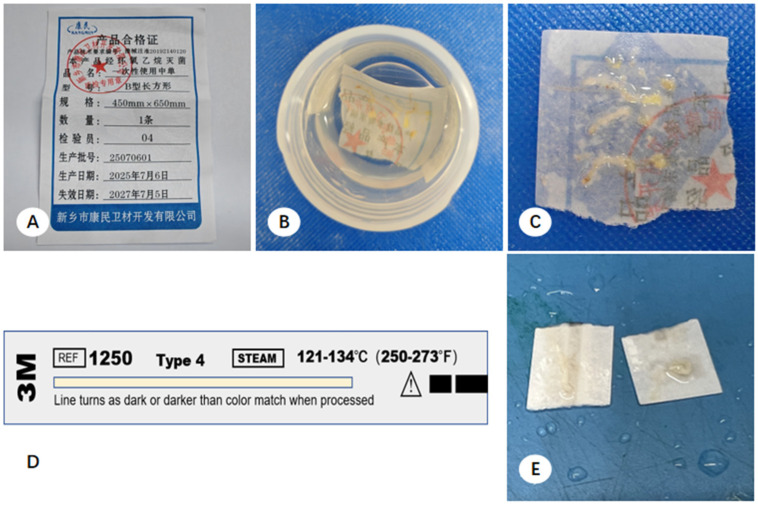
Presentation and processing of core needle biopsy (CNB) specimens. (**A**) Ethylene oxide (EO) sterilization slip from the biopsy procedure kit. (**B**) Initial state of the specimen upon arrival; CNB tissue strips are adhered to segments of the sterilization slip. (**C**) Status of the biopsy tissue on the sterilization slip segments during pathological processing; tissue presents as either intact cores or fragmented segments. (**D**) A 3M sterilization indicator card. (**E**) Segments of the 3M sterilization indicator card utilized for CNB tissue adherence.

Multicenter observations indicated that, unlike the bronchoscopy units, Interventional Radiology (IR) departments generally did not include standardized adherence materials in their sterile puncture kits. Consequently, clinicians often improvised by using product qualification certificates (from ethylene oxide-sterilized kits) or 3M steam sterilization indicator cards (from autoclaved kits) to secure CNB specimens (Figure 2). These cards—intended solely for verifying sterilization efficacy—were immersed with the tissue in 10% formalin. Gross pathological assessment revealed that these cards lost structural integrity and shed fibers upon saturation, causing them to adhere aggressively to the biopsy cores. Attempts to separate the tissue using tweezers frequently resulted in specimen fragmentation and loss of diagnostic material. Additionally, because CNB specimens often contained necrotic or friable regions. These samples frequently disintegrated into minute particles that remained suspended in the fixative, mirroring the losses observed in TBLB processing.

**Figure 3:**
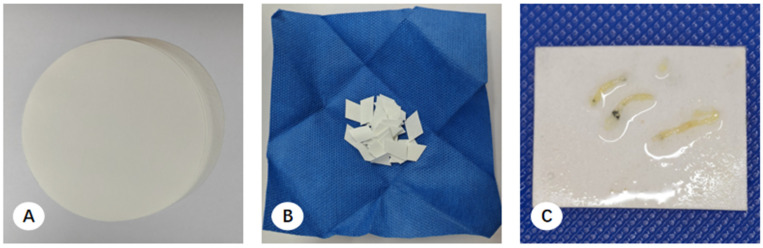
Preparation workflow of polypropylene microporous membrane (PPMM, Taiyuan Yuanyin Biotechnology Co., Ltd., Taiyuan, Shanxi, China) segments and specimen status. (**A**) Macroscopic appearance of the original PPMM. (**B**) PPMM preparation: the membrane is sectioned into 1.5 cm × 2 cm segments and wrapped in non-woven fabric for sterilization. (**C**) Specimen adherence and handling: PPMM segments are utilized for CNB tissue adherence. The tissue strips remain intact on the PPMM surface and are easily detached during gross examination.

To address these limitations, the IR department at Center A, in collaboration with the pathology department, optimized its handling protocols by utilizing pre-cut Polypropylene Microporous Membranes (PPMM). Large PPMMs (27 cm diameter) were trimmed into 1.5 cm × 2 cm rectangles, packaged, and sterilized (Figure 3). These membranes provided enhanced durability and minimal fiber shedding, thereby maintaining specimen integrity and significantly improving tissue recovery during the grossing process [14].

### 2.4. EBUS-TBNA Specimen Processing Methodologies

**Figure 4:**
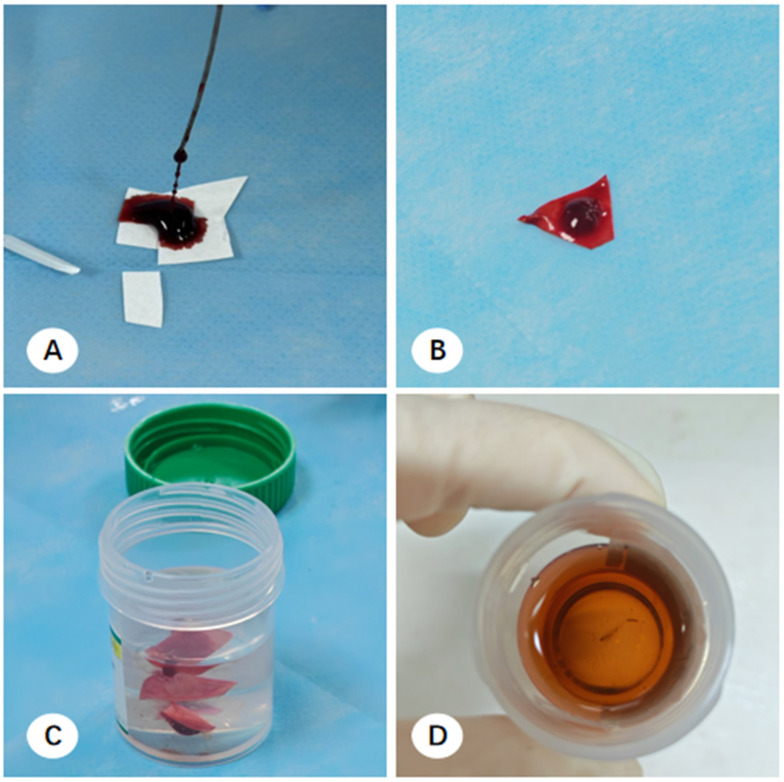
Workflow for processing EBUS-TBNA specimens using the filter paper method. (**A**) Specimen collection. Following the aspiration procedure, the staff discharge the material onto a small segment of filter paper. (**B**) Coagulation. The specimen is air-dried for 3 min to facilitate tissue coagulation and adherence. (**C**) Fixation. The filter paper is placed into a specimen container filled with formalin fixative for transport to the pathology department. (**D**) Post-processing state. Residual micro-fragments, too small for manual retrieval, remain visible in the fixative solution after the primary sample has been processed.

**Figure 5:**
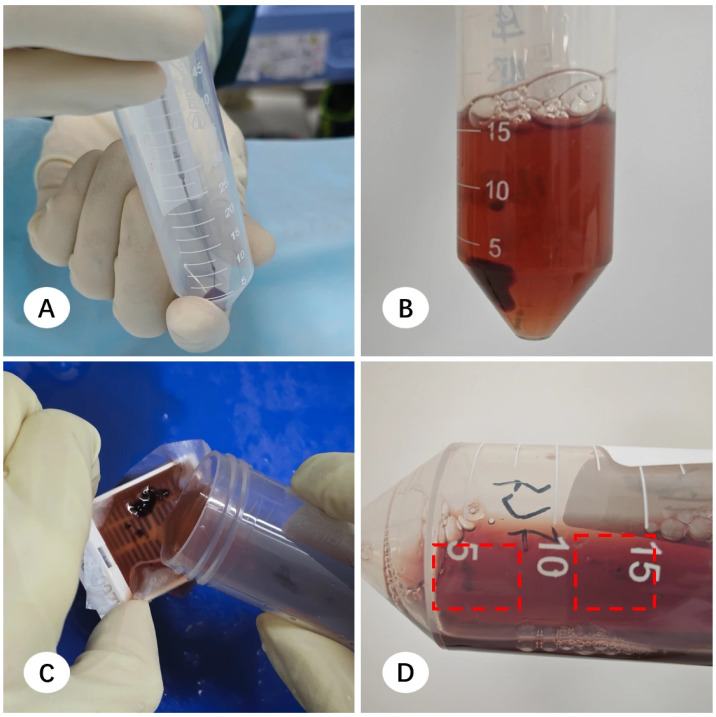
Workflow for EBUS-TBNA specimen processing using the direct self-clotting method. (**A**) Specimen expulsion. Following the aspiration procedure, the staff expel the tissue specimens onto the bottom or sidewall of a centrifuge tube. (**B**) Coagulation and fixation. Specimens are air-dried for 3 min to facilitate coagulation, after which formalin fixative is added for submission. (**C**) Sedimentary filtration. Pathology technicians utilize a decanting method; the majority of the supernatant is poured off, and the remaining sediment is filtered through embedding paper on a cassette. (**D**) Residual fragments. Following primary tissue harvest, the decanted fluid is returned to the original tube, where residual micro-fragments can still be observed (red dashed boxes indicate the regions containing residual micro-fragments).

Multicenter observations identified three primary handling methods for EBUS-TBNA specimens:

(1) Filter Paper-Based Collection: Aspirated material was expressed from the needle via a wire stylet onto pre-cut filter paper to form a coagulated mixture of tissue and blood. The resulting clot was air-dried for 3 min to ensure adequate coagulation and prevent the rapid dispersion of cellular components upon immersion. The clot and filter paper were then submerged in 10% formalin for transport (Figure 4) [16].
(2) Direct Self-clotting in Centrifuge Tubes: Aspirated materials were expelled directly into a centrifuge tube to allow for spontaneous coagulation, followed by the addition of 10% formalin fixative (Figure 5) [17].
(3) Direct Fixation: Some pulmonologists bypassed coagulation entirely, expelling the aspirated material directly into a specimen bottle containing 10% formalin for immediate stabilization.

In the pathology laboratory, processing varied based on the collection method. For filter paper-based samples, tissue clots were scraped from the paper, and macroscopically visible flakes suspended in the fixative were retrieved. These were wrapped in embedding paper and processed into FFPE cell blocks (CBs) (Figure 4). For direct self-clotting or direct fixation samples, technicians used tweezers to retrieve thread-like material or utilized a decanting method, where the majority of the liquid was poured out and the remaining sediment was filtered through embedding paper (Figure 5).

**Figure 6:**
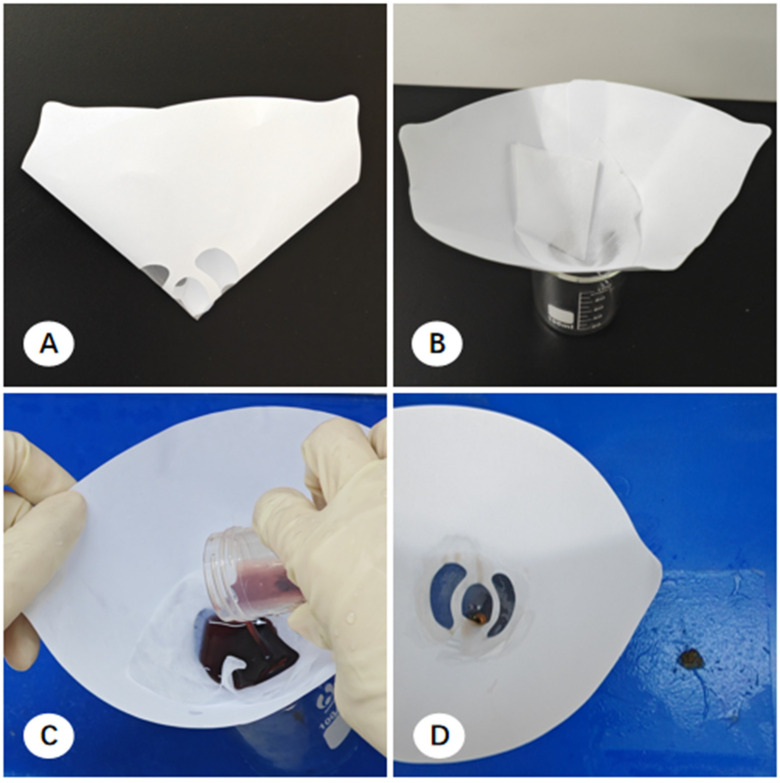
Workflow for EBUS-TBNA specimen processing using the funnel filtration method. (**A**) Funnel design. Lateral view of the paper funnel used for filtration, featuring a filter paper body and a 100-mesh nylon mesh base. (**B**) Filtration setup. The funnel is placed onto a beaker and lined with a piece of lens paper (embedding paper). (**C**) Gravity filtration. Needle aspirates within the centrifuge tube are gently agitated and poured directly into the funnel without prior centrifugation. (**D**) Harvested material. Aspirated tissue fragments remain on the lens paper surface following the completion of the filtration process.

To optimize sample recovery, the pathology technician at Center A implemented a Funnel Filtration Technique [18]. A specialized funnel device was constructed using a disposable paper funnel (Beijing Ruimiao Jingyi Trading Co., Ltd., Beijing, China), which was originally de-signed for paint filtration, with a fine-mesh base. A piece of embedding paper was placed inside the funnel, which was positioned over a beaker. The solid–liquid mixture from the centrifuge tube was poured through this device, allowing the fixative to drain rapidly while ensuring that all tissue clots and dispersed cellular material were captured on the lens paper. This collected material was then wrapped and processed into CBs using the standard FFPE protocol (Figure 6).

### 2.5. FFPE Specimen Processing and Pre-Analytic Assessment

All small biopsy specimens were processed on the day of laboratory receipt. Following gross assessment, formalin-fixed biopsy fragments were retrieved, wrapped in embedding paper, and placed in cassettes for paraffin embedding. The next day, FFPE blocks were sectioned at 4 μm and stained with H&E. Upon histopathological confirmation of NSCLC, the total tumor cell count and the neoplastic cell fraction were quantified for each section. Samples were classified as adequate for molecular testing if they contained more than 200 tumor cells per section and exhibited a tumor cell fraction above the established thresholds (typically >10% or >5%, depending on the specific molecular assay requirements). A specimen was deemed adequate only if it simultaneously met both criteria: having at least 200 tumor cells per section and a tumor cell fraction at or above the aforementioned thresholds.

### 2.6. Cell Pellet Collection and Cellularity Evaluation

During the final two months of the study, a modified protocol was implemented to salvage residual cellular material from the fixative solution. On the day of gross processing, following primary tissue retrieval, pathology technicians retained the residual fixative while discarding the adherence paper. For EBUS-TBNA specimens, the supernatant was preserved in a secondary centrifuge tube during decanting. Once the primary tissue was harvested, the supernatant was returned to the original tube for storage. The next day, in cases where NSCLC was confirmed but the FFPE tissue was deemed inadequate for molecular profiling through pre-analytic assessment, the residual fixative was processed to generate a cell pellet. The recovery technique for EBUS-TBNA samples varied by site according to local harvesting practices: Center A: Utilized a funnel filtration method; because this process sequestered all viable cellular material, the filtered residual solution was devoid of diagnostic cells and was not collected. Centers B and C: The supernatant was preserved and the residual fixative was centrifuged to harvest a cell pellet for further evaluation.

The residual medium was transferred to a 15 mL tube and centrifuged at 1000× *g* for 5 min. The resulting pellets were resuspended in 1 mL of phosphate-buffered saline (PBS), transferred to a 1.5 mL microcentrifuge tube, and centrifuged at 3000× *g* for 5 min. To mitigate nucleic acid crosslinking caused by prolonged formalin exposure, the pellets underwent two additional PBS wash cycles. Following the final wash and supernatant removal, the pellets were resuspended in 250 μL of PBS. A 50 μL aliquot was used to prepare a direct smear, which was air-dried and H&E-stained. The remaining 200 μL suspension was centrifuged at 3000× *g* for 5 min; the supernatant was then discarded, and the resulting cell pellet was stored at −80 °C for DNA extraction [14,15,18]. For cases requiring molecular testing of residual medium, cell pellet harvesting is typically completed within 48 h of sample receipt by the laboratory.

Neoplastic cellularity was quantified by scanning the index smear via light microscopy in a systematic raster pattern. Because the final stored pellet represented four times the volume of the 50 μL aliquot, molecular adequacy was extrapolated from the smear. Samples were classified as adequate if the index smear contained more than 50 tumor cells (corresponding to >200 cells in the final pellet) and a tumor fraction of >10%.

For cell pellet samples deemed adequate by cell counting evaluation, DNA was extracted using the QIAGEN DNeasy Blood and Tissue Kit (Qiagen, Hilden, Germany) following the manufacturer’s protocol. The extracted DNA was quantified with a Qubit 2.0 fluorometer (Invitrogen, Carlsbad, CA, USA). Samples were required to contain >10 ng of DNA for PCR analysis and >30 ng for NGS testing.

### 2.7. Statistical Analysis

Statistical analyses were performed using SPSS version 26.0 (IBM Corp., Armonk, NY, USA). Descriptive statistics were used to summarize specimen characteristics and baseline adequacy rates. To evaluate the baseline consistency across the three participating centers, Pearson’s chi-squared test (with 2 degrees of freedom, X2critical value = 5.991) was employed for inter-institutional comparisons of each biopsy modality (TBLB, CNB, and EBUS-TBNA). To control for the potential inflation of Type I error due to multiple comparisons between the three centers (Center A vs. B, A vs. C, and B vs. C), the Bonferroni correction was pre-specified, resulting in an adjusted significance threshold of α’ = (0.05/3).

For the assessment of adequacy improvement following the addition of cell pellets, each specimen served as its own internal control. The significance of the incremental adequacy was analyzed using McNemar’s test for paired categorical data. Specifically, the McNemar chi-square asymptotic formula was used when the number of discordant pairs (b + c) was ≥25; for instances where b + c < 25, the Binomial exact test was applied to ensure statistical accuracy. *p*-value < 0.05 was considered statistically significant.

## 3. Results

### 3.1. Patient Clinical Characteristics

During the six-month study period, 3705 pathology samples were collected from patients diagnosed with non-small cell lung cancer (NSCLC) via minimally invasive biopsy. The cohort comprised 2438 males and 1267 females, with a median age of 64 years (range: 14–93 years). Diagnostic procedures included TBLB (*n* = 1334), CNB (*n* = 1490) and EBUS-TBNA (*n* = 881). All cases were confirmed as NSCLC through H&E staining of FFPE sections and IHC analysis. Histological subtypes included adenocarcinoma (*n* = 2278), squamous cell carcinoma (*n* = 933), and NSCLC-not otherwise specified (NSCLC-NOS; *n* = 494) (Table 1).

### 3.2. FFPE Tissue Pre-Analytic Assessment for Molecular Testing

Using a conventional adequacy threshold—defined as >200 tumor cells per section and a tumor cell fraction > 10% (Criterion A)—the overall adequacy rate of FFPE samples for the 3705 cases was 80.6% (2986/3705). Individual adequacy rates varied by procedure: 84.7% for CNB, 86.4% for EBUS-TBNA, and 72.2% for TBLB. However, given the enhanced sensitivity of contemporary molecular platforms, which can often detect mutations at a tumor cell fraction as low as 1%, we also evaluated a refined adequacy standard (>200 tumor cells and a tumor cell fraction > 5%) (Criterion B). Under this adjusted criterion, the overall adequacy rate increased to 88.9% (3293/3705). Subgroup analysis showed improved adequacy across all procedures, with rates of 91.4% for CNB, 89.1% for EBUS-TBNA, and 85.9% for TBLB. The performance of individual biopsy methods at each institution is detailed in Table 2. Inter-institutional comparisons revealed no significant differences in adequacy rates across the three participating centers (*p* > 0.05; Table 2). Statistical analysis showed that under Criterion A, the *p*-values for inter-institutional consistency were 0.164 for TBLB, 0.389 for CNB, and 0.787 for EBUS-TBNA. Under Criterion B, the *p*-values were 0.143 for TBLB, 0.698 for CNB, and 0.815 for EBUS-TBNA. As all initial global comparisons across the three institutions yielded *p* > 0.05, it was concluded that there were no significant inter-institutional differences. Consequently, no further post hoc pairwise comparisons were required (Table 2).

Eighty patients underwent both TBLB and EBUS-TBNA. For these cases, molecular testing requirements were considered met if at least one sample type was adequate. The combined adequacy rates at Center A, Center B, and Center C were 91.3% (53/58), 100% (3/3), and 94.7% (18/19), respectively (Table 3).

### 3.3. Microscopic Characteristics and Molecular Adequacy of Residual Fixative-Derived Cell Pellets

**Figure 7:**
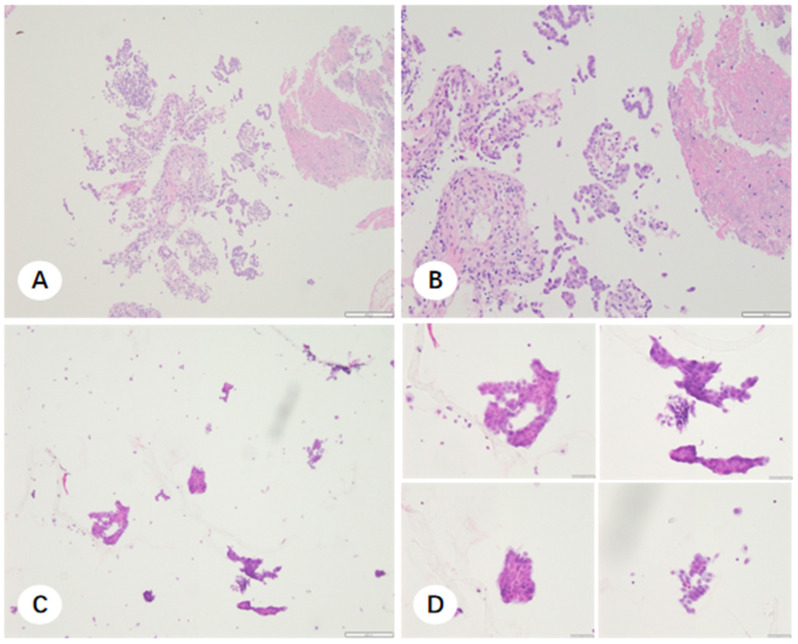
Paraffin-embedded tissue sections and corresponding fixative cell sediment smears from an unsatisfactory TBLB specimen. (**A**,**B**) Hematoxylin and eosin (H&E) stained paraffin sections showing scant neoplastic tissue, with minute tumor fragments adjacent to normal tissue ((**A**) ×100; (**B**) ×200). (**C**,**D**) Corresponding cell sediment smears from the same case exhibiting abundant, diagnostic tumor cells ((**C**) ×100; (**D**) ×400).

**Figure 8:**
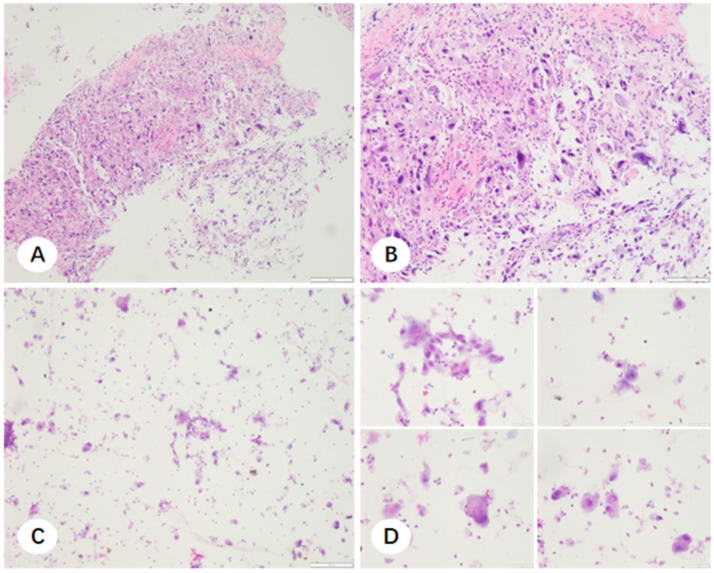
Paraffin-embedded tissue sections and corresponding fixative cell sediment smears from a CNB specimen. (**A**,**B**) H&E-stained paraffin sections demonstrating extensive tumor necrosis and loosely arranged neoplastic cells ((**A**) ×100; (**B**) ×200). (**C**,**D**) Corresponding cell sediment smears containing high tumor cellularity ((**C**) ×100; (**D**) ×400).

**Figure 9:**
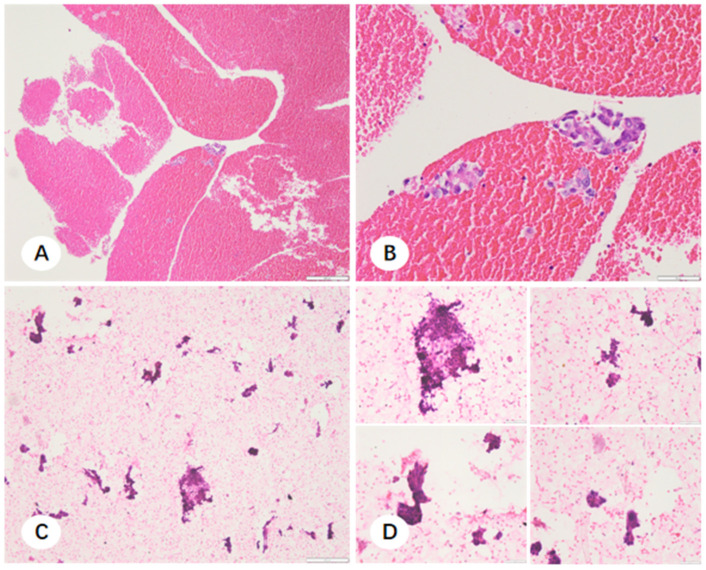
Paraffin-embedded tissue sections and corresponding fixative cell sediment smears from EBUS-TBNA specimens (filter paper method). (**A**,**B**) Paraffin sections displaying limited tumor tissue representation ((**A**) ×100; (**B**) ×200). (**C**,**D**) Corresponding cell sediment smears exhibiting abundant tumor cells ((**C**) ×20; (**D**) ×100).

**Figure 10:**
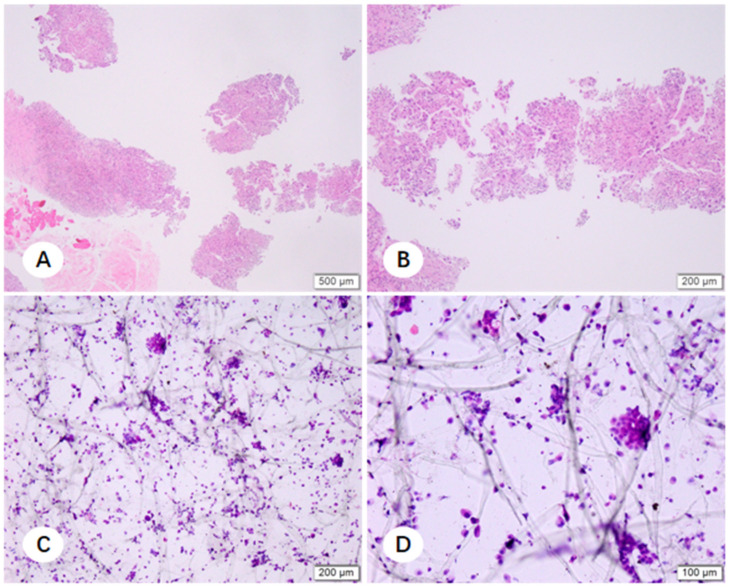
CNB specimens collected using 3M sterilization indicator cards and corresponding sediment smears. (**A**,**B**) H&E-stained paraffin sections showing fragmented tumor tissue and dissociated neoplastic cells ((**A**) ×40; (**B**) ×100). (**C**,**D**) Corresponding cell sediment smears containing abundant tumor cells interspersed with paper fibers ((**C**) ×100; (**D**) ×200).

In cases where NSCLC was histologically confirmed but the FFPE sections were insufficient for molecular profiling, residual fixative was processed into cell pellets. Morphological evaluation revealed that these cell pellet smears frequently exhibited excellent tumor cellularity, particularly in instances where the corresponding FFPE sections were compromised by tissue fragility or necrosis. The comparative microscopic features of FFPE sections and cell pellet smears for TBLB, CNB, and EBUS-TBNA are illustrated in Figure 7, Figure 8 and Figure 9. Notably, cell pellet smears derived from CNB samples utilizing steam sterilization indicator cards as adherence material contained numerous thick, elongated paper fibers (Figure 10). The presence of these disordered fibers resulted in uneven staining and obscured critical cytomorphological details.

During the final two months of the study, the baseline adequacy rates for TBLB and CNB across the three centers were 75.2% (200/266) and 84.3% (375/445), respectively. Among the cases with inadequate FFPE cellularity, the salvage of residual fixative yielded adequate cell pellet samples in 59.1% (39/66) of TBLB cases and 48.6% (34/70) of CNB cases. Consequently, the combined adequacy rate increased to 89.8% (239/266) for TBLB and 91.9% (409/445) for CNB. Statistical analysis showed that the improvement was highly significant for TBLB at Center A and C (both *p* < 0.001), as well as for CNB at Center A (*p* = 0.0039), Center B (*p* = 0.0313), and Center C (*p* < 0.001). For EBUS-TBNA samples at Centers B and C, the initial adequacy rate was 84.2% (16/19) and 82.6% (71/86) respectively. Recovering the supernatant from cases with inadequate FFPE cellularity provided adequate cell pellets in 66.7% (2/3) and 73.3% (11/15) of instances. The combined adequacy rate increased to 94.7% (18/19) for Center B and 95.3% (82/86) for Center C. Statistical analysis showed that a significant increment was observed at Center C (*p* < 0.001). Detailed performance metrics for each biopsy method by institution are provided in Table 4.

### 3.4. DNA Yield and Molecular Testing Performance from Cell Pellet Samples

DNA was extracted from 86 cell pellet samples deemed adequate by cell counting evaluation. The median DNA yield was 249 ng (range: 53–2532 ng), with all samples surpassing the threshold required for Next-Generation Sequencing (NGS). According to the clinical protocol at Center A, cell pellets are processed for sequencing when the corresponding FFPE tissue specimen is deemed inadequate for molecular analysis. During the final two months of the study, cell pellets from 10 eligible cases were used as substitutes for FFPE tissue. Somatic mutations were identified in all 10 cases, five of which harbored clinically actionable mutations. Detailed genetic profiles for these cases are provided in Table 5. As the sequencing success rate of cell pellet samples in Centers B and C has not yet been verified, comprehensive workflow validation and inter-institutional evaluation are required before this protocol can be standardized across all partner sites.

## 4. Discussion

In the current landscape of precision oncology, the management of lung cancer relies heavily on obtaining sufficient neoplastic material for both definitive histological subtyping and comprehensive molecular profiling. As targeted therapies and immunotherapies expand, the “tissue is issue” challenge has intensified; small biopsy specimens obtained via minimally invasive techniques—such as image-guided CNB or EBUS-TBNA—frequently face the risk of tissue exhaustion. Consequently, optimizing the processing of these limited specimens has become a critical priority to ensure that every patient receives a complete molecular diagnosis from a single invasive procedure [4,5,6,7,8,9,10].

Strategic optimization involves a multi-modal approach to specimen handling that shifts away from traditional, tissue-depleting workflows. A cornerstone of this modern strategy is the salvage of “medical waste” substrates, including rinsing the coaxial or aspiration needle and harvesting cell pellets from the residual transport fixative [14,15,18,19,20,21,22]. These fluids often contain high-quality exfoliated cells and tissue fragments that, when processed into cell pellet, cell blocks or liquid-based cytology (LBC) slides, provide an essential secondary reservoir of tumor cells. These adjuncts not only increase diagnostic sensitivity—particularly in cases where the primary solid core is necrotic or scant—but also offer a high-yield source of high-quality nucleic acids for downstream Next-Generation Sequencing (NGS) [14,15].

Enriched tumor cells are frequently found in ancillary fluids—such as bronchial biopsy rinses, core needle rinses, and residual fixative media—due to a combination of the biological characteristics of lung cancer and the mechanical trauma of the biopsy procedure. Lung cancer tissues are often characterized by reduced cell-to-cell adhesion and structural fragility. Unlike healthy, organized tissue, malignant cells are easily dislodged from the main tumor mass. The act of inserting a needle or using biopsy forceps acts as a “mechanical scraping”. This physical disruption breaks off small clusters of tumor cells and microscopic tissue fragments from the lesion. As the needle or forceps transgresses the tumor and is withdrawn, cells often migrate or “spill” into the lumen of the needle or the surrounding fluid track. Once the solid core biopsy is placed into a transport fixative (like formalin), the mechanical trauma already sustained causes tumor cells to continue shedding into the surrounding liquid.

A study by Rosell et al. demonstrated that cytological examination of rinse fluid from bronchial biopsies increased the diagnostic yield for endobronchial malignancies by 4.8%. This technique maximizes the use of materials often discarded during bronchoscopy and adds no significant time or expense to the procedure [19]. Research by Mojica et al. suggests that salvaging cells from CNB washes can significantly extend the utility of small specimens without compromising the integrity of the primary tissue core [20]. Lan et al. (2021) further validated this approach, showing that LBC performed on core needle rinses (CNR) significantly improves diagnostic sensitivity for peripheral lung nodules [21]. By analyzing 406 patients, they found that combining CNR with traditional CNB increased sensitivity from 92.5% to 96.4%, effectively reducing false-negative rates [21].

Two additional studies explored using these alternative sample sources for genetic testing in NSCLC. Sakairi et al. (2014) investigated using saline rinse solutions from bronchoscopic biopsy needles—termed ultra-microsamples—for comprehensive biomarker testing [22]. Their analysis of 1474 molecular tests showed a 99.3% concordance rate with results from conventional histological samples [22]. A 2022 study in thoracic cancer evaluated the feasibility of using supernatants from core-needle biopsy samples as a liquid biopsy source for 48 patients with lung adenocarcinoma. The study reported a 95.8% concordance rate for DNA-level mutation detection compared to tissue samples. While the results confirm that supernatants are a valuable source for genotyping, the researchers noted that methods for preserving and extracting RNA from these specimens require further improvement [23].

Our preliminary single-center studies demonstrated the significant potential of collecting cell pellets from the residual fixative medium of both TBLB and CNB samples. Conventionally, this medium, containing tiny tissue fragments and cell clusters, is discarded. Our study found that the fixative medium was frequently enriched with tumor cells. Among 324 TBLB samples, 21.6% had inadequate FFPE tissue sections for molecular analysis; however, 75.7% of these cases yielded adequate cell pellet samples. Adequacy rates for molecular testing of CNB sample were 86.4% in FFPE samples and 92.3% in cell pellet samples. Incorporating cell pellet analysis increased overall molecular testing adequacy to 95.2%.

In this study, we investigated the adequacy rates of molecular testing for small lung cancer biopsy specimens obtained via minimally invasive procedures at our hospital and two other high-volume oncology centers in the same city. We evaluated a total of 3705 NSCLC cases diagnosed through minimally invasive techniques across these three institutions during a six-month period in 2024. Using a conventional threshold for adequacy—defined as cases containing >200 tumor cells per section and a tumor cell fraction > 10%—the overall adequacy rate was 80.6% (2986/3705). Under this adjusted standard of adequacy criteria to >200 tumor cells per section and a tumor cell fraction > 5%. the overall adequacy rate in our cohort increased to 88.9% (3293/3705). Nevertheless, a subset of specimens remains insufficient for molecular analysis, suggesting that there is still significant room for improvement in specimen handling protocols and laboratory workflows.

During the final two months of the study, we applied our previously established single-center optimization strategy for small specimens to those that remained inadequate for molecular testing. Specifically, we collected cell sediment from the residual fixative after tissue sampling to prepare cell smears, which were then evaluated for tumor cell quantity and adequacy. Among the 154 cases collected, 86 met the requirements for molecular testing. By combining paraffin sections with cell sediment analysis, the overall adequacy rate for molecular testing was successfully improved from 81.1% to 91.7%.

Our preliminary observations identified specific nuances in specimen collection techniques within the bronchoscopy and interventional radiology (IR) departments that adversely affect downstream processing in pathology. These issues were prevalent across all three participating hospitals. In most IR departments, sterile puncture kits lack pre-cut filter paper for tissue adhesion. Consequently, clinicians often improvise by using product qualification certificates (from ethylene oxide sterilization) or 3M steam sterilization indicator cards (from autoclaves) to secure CNB specimens.

Gross pathological assessment revealed that these indicator cards soften and lose structural integrity—frequently shedding fibers—once saturated with fixative. This practice complicates the routine collection of small tissue samples for paraffin sectioning and hinders the recovery of residual cell sediments. To address this, our hospital’s IR and pathology departments collaborated to optimize handling by using sterilized, pre-cut PPMM segments. This transition has improved tissue recovery and specimen integrity while minimizing fiber contamination.

Aspirated materials from EBUS-TBNA can be processed as liquid-based cytology, direct smears, or cell blocks (CBs) [2]. CB preparation is particularly critical, as it allows for superior morphological evaluation, IHC staining, and molecular testing. However, unlike surgical histology, CB preparation lacks a standardized protocol; a 2014 survey identified over ten different methods, each with distinct advantages. This lack of standardization contributes to practical challenges, as evidenced by the 44% of cytopathologists who express dissatisfaction with the quality of CB specimens [24]. The “tissue coagulum clot” (TCC) and “self-clotting” (SC) techniques are alternatives designed to improve cellular yield over conventional saline rinsing. In the TCC method, aspirated material is expelled onto filter paper [16], a technique that has demonstrated a diagnostic yield of 88.7% compared to 56.4% for saline rinsing. In the SC method, material is expelled directly into a centrifuge tube to air-dry before formalin fixation [17]. While filter paper-based TCC improves EBUS-TBNA yields, it can impede downstream pathology workflows. Consequently, our bronchoscopy unit has transitioned to “direct self-clotting” in centrifuge tubes without filter paper. Simultaneously, the pathology department optimized collection using a funnel filtration method to ensure that all tissue clots and dispersed cellular materials are harvested [18].

Observations at the other two participating hospitals revealed inconsistent collection methods, with some utilizing filter-paper-based TCC and others using the SC technique. During harvesting, technicians typically used tweezers for large fragments or decanting and filtration for smaller sediments. These manual processes are highly susceptible to specimen loss; tweezers often leave residual tumor cells in the vial, while decanting risks the accidental disposal of suspended material. Notably, during the final two months of this study, we confirmed that in cases where initial CB sections had insufficient cellularity, recovering the residual fixative or decanted supernatant often yielded enough tumor cells for molecular testing.

The persistence of these suboptimal practices at Centers B and C—specifically the use of non-validated adherence materials (such as 3M sterilization indicator cards) for CNB specimens and decanting-based workflows for EBUS-TBNA samples—constitutes a widespread, systemic issue in the pre-analytical phase of diagnostics. We strongly advocate for a coordinated effort between the Society of Interventional Radiology (SIR), the College of American Pathologists (CAP), and the International Association for the Study of Lung Cancer (IASLC) to establish and enforce standardized technical protocols. Specifically, clinical societies should issue standards mandating that sterile core needle biopsy (CNB) kits incorporate validated, medical-grade, and fixative-resistant materials—such as the PPMM segments successfully piloted in our study. Integrating these materials into the manufacturing of pre-packaged kits would shift the responsibility for specimen integrity from ad hoc clinical improvisation to a standardized, manufacturer-validated process. This transition is essential to eliminate fiber contamination, preserve tissue architecture during fixation, and ultimately ensure that the limited tissue obtained via CNB is fully optimized for downstream genomic analyses. For EBUS-TBNA and other fine needle aspiration (FNA) procedures, we advocate for the development and adoption of specialized commercial products. These products should be designed to efficiently filter out excess fixative media while ensuring maximal retention of cellular material for diagnostic and molecular studies.

In a previous study, we compared sequencing data from 190 NSCLC patients who underwent both tissue-based next-generation sequencing (tissue-NGS) and plasma-based NGS (plasma-NGS). The overall concordance rate between tissue and plasma samples was 78.9% (150/190). The sensitivity of tissue-NGS and plasma-NGS was 95.0% and 71.9%, respectively. The suboptimal sensitivity of plasma-NGS was attributed to the fact that some tumors do not shed ctDNA into the bloodstream or shed it at levels too low to be detected [11]. We also validated the concordance of variant detection between FFPE tissue sections and matched cell pellet samples, confirming that somatic mutations identified in FFPE sections were consistently detected in corresponding cell pellets [14,15]. Furthermore, we compared the molecular testing performance of plasma and cell pellet samples. In 45 cases with insufficient FFPE tissue, both plasma-based and cell pellet-based NGS were performed. All samples were successfully sequenced, and targetable mutations found in plasma were also identified in matched cell pellets. Notably, in four cases, targetable mutations were detected only in cell pellets—highlighting the superior sensitivity of cell pellets over plasma for molecular testing [14].

Given that collecting cell pellets is neither time-consuming nor costly, and imposes no additional burden or risk on patients, we recommend the following clinical workflow: in cases with insufficient FFPE tissue, cell pellet samples should be prioritized for sequencing when available. Plasma should be used only if adequate cell pellet material cannot be obtained. Moreover, any negative plasma result should be followed by a repeat tissue biopsy to avoid false negatives and ensure that actionable driver mutations are not missed.

In the clinical workflow at Center A, tumor cellularity in NSCLC small biopsy FFPE sections is routinely evaluated and documented in the diagnostic report. For cases where NSCLC is confirmed, but the FFPE tissue is deemed inadequate for molecular profiling upon pre-analytical review, the residual specimen fixative is subsequently processed to generate both an index smear and a cell pellet. A cytology diagnosis is issued based on the index smear, stating the tumor cellularity and its adequacy for molecular analysis. The matching cell pellet sample that is determined to be adequate then serves as a substitute for sequencing.

This study has several limitations. Factors such as operator experience, number of needle passes, and needle size were not standardized across participating institutions, which may have affected the cellular yield of biopsy specimens. These are major confounding variables that could largely account for the observed inter-center differences. Nevertheless, the primary objective of this study was to identify potential areas for improving molecular adequacy in NSCLC diagnostic samples under such real-world variables, and to evaluate the value of implementing residual fixative-derived cell pellets as a supplementary source for molecular profiling.

While pathology laboratories can control the post-receipt processing timeline—typically ensuring cell pellet harvesting within 48 h—the total formalin fixation time is variable, depending on the time elapsed from biopsy acquisition to laboratory arrival. This pre-analytical variability poses a risk to DNA quality in the resulting cell pellets. Therefore, to maintain nucleic acid integrity and ensure reliable downstream results, clinical workflows should adhere to appropriate fixation times, ideally between 6 and 72 h, for small biopsy specimens intended for molecular testing.

Additionally, only 10 eligible cell pellet cases from Center A were selected for sequencing, based on DNA yield thresholds and clinical demand; therefore, the reproducibility of these DNA yield results and sequencing success rates at Centers B and C was not confirmed. In those centers, suboptimal adherence materials (3M sterilization indicator cards) and decanting-based workflows were still in use. The potential impact of this inter-center variability in pre-analytical methods represents a recognized constraint of the current molecular dataset, underscoring the need for standardized protocol implementation and subsequent multi-center validation.

## 5. Conclusions

Despite the increased sensitivity of modern molecular platforms, a subset of specimens remains insufficient for analysis. Strategic optimization requires a multimodal approach to specimen handling. First, clinical support staff must use appropriate materials and containers during minimally invasive biopsies to facilitate pathology processing. Second, the pathology department must ensure that all tissue fragments and cellular debris are fully utilized. Retrieving supplemental tumor cells from residual fixative can provide life-changing molecular testing opportunities for patients whose initial paraffin-embedded sections were deemed inadequate.

## Figures and Tables

**Table 1:** Patient Clinical Characteristics.

Patient Characteristics (*N* = 3705)	No. of Patients (%)
**Age**	
Median: 64	
Range: 14–93	
**Gender**	
Male:	2438 (65.8%)
Female:	1267 (34.2%)
**Diagnosis**	
Adenocarcinoma	2278 (61.5%)
Squamous cell carcinoma	933 (25.2%)
NSCLC not otherwise specified	494 (13.3%)
**Detecting methods**	
CNB	1490 (40.2%)
EBUS-TBNA	881 (23.8%)
TBLB	1334 (36.0%)

**Table 2:** Adequacy Rates of Three Small Biopsy Types for NSCLC Molecular Testing in Three Hospitals.

Biopsy Types	Hospitals	Adequacy Rate ^A^	Adequacy Rate ^B^
TBLB	A	71.6% (724/1011)	85.7% (866/1011)
	B	78.1% (114/146)	90.4% (132/146)
	C	70.6% (125/177)	83.6% (148/177)
	Average	72.2% (963/1334)	85.9% (1146/1334)
CNB	A	85.9% (561/653)	91.1% (595/653)
	B	84.9% (276/325)	90.8% (295/325)
	C	83.0% (425/512)	92.2% (472/512)
	Average	84.7% (1262/1490)	91.4% (1362/1490)
EBUS-TBNA	A	87.1% (499/573)	89.9% (515/573)
	B	84.6% (115/136)	87.5% (119/136)
	C	85.5% (147/172)	87.8% (151/172)
	Average	86.4% (761/881)	89.1% (785/881)
Total	Average	80.6% (2986/3705)	88.9% (3293/3705)

^A^, with >200 tumour cells/section and a tumour cell fraction ≥ 10%; ^B^, with >200 tumour cells/section and a tumour cell fraction ≥ 5%. Note: 95% CI, 95% confidence interval, calculated using the Wilson score method.

**Table 3:** Combined adequacy rates for patients undergoing both TBLB and EBUS-TBNA.

Hospitals	TBLB Adequacy Rate	EBUS-TBNA Adequacy Rate	Cases with at Least One Sample Adequate
A	69.0% (40/58)	84.5% (49/58)	91.3% (53/58)
B	100% (3/3)	33.3% (1/3)	100% (3/3)
C	63.2% (12/19)	89.4% (17/19)	94.7% (18/19)
Average	68.8% (55/80)	83.8% (67/80)	92.5% (74/80)

**Table 4:** Increases in adequacy rates by adding adequate cell pellets.

Biopsy Types	Hospitals	Adequacy Rate ^A^	Combined Adequacy Rate by Adding Adequate Cell Pellet	Adequacy Rate ^B^	Combined Adequacy Rate by Adding Adequate Cell Pellet
TBLB	A	75.3% (134/178, 95% CI: 68.4–81.1%)	90.4% (161/178, 95% CI: 87.9–95.7%)	78.7% (140/178, 95% CI: 72.1–84.1%)	94.4% (168/178, 95% CI: 90.0–97.0%)
	B	78.6% (22/28, 95% CI: 60.0–90.0%)	89.3% (25/28, 95% CI: 72.8–96.3%)	82.1% (23/28, 95% CI: 64.4–92.1%)	92.9% (26/28, 95% CI: 77.4–98.0%)
	C	73.3% (44/60, 95% CI: 61.0–82.9%)	88.3% (53/60, 95% CI: 77.8–94.2%)	73.3% (44/60, 95% CI: 64.6–85.6%)	90% (54/60, 95% CI: 79.9–95.3%)
	Average	75.2% (200/266, 95% CI: 69.6–80.1%)	89.8% (239/266, 95% CI: 85.6–93.0%)	77.8% (207/266, 95% CI: 72.4–82.5%)	93.2% (248/266, 95% CI: 89.6–95.7%)
CNB	A	84.2% (101/120, 95% CI: 76.5–89.7%)	91.7% (110/120, 95% CI: 85.3–95.5%)	86.7% (104/120, 95% CI: 79.4–91.7%)	92.5% (111/120, 95% CI: 86.3–96.0%)
	B	86.4% (76/88, 95% CI: 77.6–92.1%)	93.2% (82/88, 95% CI: 85.9–96.9%)	90.9% (80/88, 95% CI: 83.0–95.3%)	93.2% (82/88, 95% CI: 85.9–96.9%)
	C	83.5% (198/237, 95% CI: 78.3–87.8%)	91.7% (217/237, 95% CI: 87.3–94.6%)	87.3% (207/237, 95% CI: 82.5–91.0%)	92.4% (219/237, 95% CI: 88.3–95.2%)
	Average	84.3% (375/445, 95% CI: 80.6–87.4%)	91.9% (409/445, 95% CI: 89.0–94.1%)	87.9% (391/445, 95% CI: 84.5–90.6%)	92.6% (412/445, 95% CI: 89.8–94.8%)
EBUS-TBNA	B	84.2% (16/19, 95% CI:62.4–94.5%)	94.7% (18/19, 95% CI: 75.4–99.1%)	89.5% (17/19, 95% CI: 68.6–97.1%)	94.7% (18/19, 95% CI: 75.4–99.1%)
	C	82.6% (71/86, 95% CI: 73.3–89.1%)	95.3% (82/86, 95% CI: 88.7–98.2%)	84.9% (73/86, 95% CI: 75.9–90.9%)	95.3% (82/86, 95% CI: 88.7–98.2%)
	Average	82.9% (87/105, 95% CI: 74.5–88.9%)	95.2% (100/105, 95% CI: 89.3–98.0%)	85.7% (90/105, 95% CI: 77.7–91.2%)	95.2% (100/105, 95% CI: 89.3–98.0%)
Total	Average	81.1% (662/816, 95% CI: 78.3–83.6%)	91.7% (748/816, 95% CI: 89.6–93.4%)	84.3% (688/816, 95% CI: 81.7–86.7%)	93.1% (760/816, 95% CI: 91.2–94.7%)

^A^, with >200 tumour cells/section and a tumour cell fraction ≥ 10%; ^B^, with >200 tumour cells/section and a tumour cell fraction ≥ 5%; Note: 95% CI, 95% confidence interval, calculated using the Wilson score method.

**Table 5:** NGS results from cell pellet samples.

Case	Diagnosis	DNA Yield (ng)	Mutation Detected by NGS
1	Squamous cell carcinoma	89.2	PIK3CA EX5 N345K:6.3%
2	Adenocarcinoma	62.5	TP53 EX7 E258V:7.2%
3	Adenocarcinoma	159.2	TP53 EX5 Q136 *:19.4% RB1 EX9 V306Tfs *12:15.5%
4	Squamous cell carcinoma	102.8	CDKN2A EX2 L78Hfs *41:74.8% TP53 EX8 R283.Afs *62:73.0% MYC amplification:4.5% PIK3CA amplification:4.4%
5	Adenocarcinoma	255.1	EGFR EX21 L858R:71.6% CDK4 amplification:8.1% MYC amplification:7.7% EGFR amplification:5.3%
6	Squamous cell carcinoma	115.0	TP53 EX10 R337L:8.6% PIK3R1 EX11 D464_L466del:6.0%
7	NSCLC not otherwise specified	73.9	EGFR EX19 E746:46.3% TP53 EX6 E204:32.5%
8	Adenocarcinoma	58.5	EGFR EX21 L858R:56.7%
9	Adenocarcinoma	141.7	EGFR EX21 L858R:54.6% STK11 EX5 W239C:66.2% MCL1 amplification:6.1% MYC amplification:6.1%
10	Adenocarcinoma	365.2	EGFR EX18 G719A:10.3% TP53 EX4 A86 *:14.3%

The asterisk (*) represents a Premature Stop (Nonsense Mutation) or Frameshift Termination. EX: The “Exon”. VAF: The Variant Allele Frequency.

## Data Availability

The original contributions presented in this study are included in the article. Further inquiries can be directed to the corresponding author.
